# Covalent Decoration of Cortical Membranes with Graphene Oxide as a Substrate for Dental Pulp Stem Cells

**DOI:** 10.3390/nano9040604

**Published:** 2019-04-12

**Authors:** Roberta Di Carlo, Susi Zara, Alessia Ventrella, Gabriella Siani, Tatiana Da Ros, Giovanna Iezzi, Amelia Cataldi, Antonella Fontana

**Affiliations:** 1 Department of Pharmacy, University “G. d’Annunzio”, Via dei Vestini, 66100 Chieti, Italy; roberta.dicarlo@unich.it (R.D.C.); susi.zara@unich.it (S.Z.); alessia.ventrella@unich.it (A.V.); gabriella.siani@unich.it (G.S.); amelia.cataldi@unich.it (A.C.); 2 Department of Chemical and Pharmaceutical Sciences, University of Trieste, Piazzale Europa 1, 34127 Trieste, Italy; daros@units.it; 3 Department of Medical, Oral and Biotechnological Sciences, University “G. d’Annunzio”, Via dei Vestini, 66100 Chieti, Italy; giovanna.iezzi@unich.it

**Keywords:** graphene oxide, covalent functionalization, cortical membranes, calcium phosphate deposition

## Abstract

(1) Background: The aim of this study was to optimize, through a cheap and facile protocol, the covalent functionalization of graphene oxide (GO)-decorated cortical membrane (Lamina^®^) in order to promote the adhesion, the growth and the osteogenic differentiation of DPSCs (Dental Pulp Stem Cells); (2) Methods: GO-coated Laminas were fully characterized by Scannsion Electron Microscopy (SEM) and Atomic Force Microscopy (AFM) analyses. In vitro analyses of viability, membrane integrity and calcium phosphate deposition were performed; (3) Results: The GO-decorated Laminas demonstrated an increase in the roughness of Laminas, a reduction in toxicity and did not affect membrane integrity of DPSCs; and (4) Conclusions: The GO covalent functionalization of Laminas was effective and relatively easy to obtain. The homogeneous GO coating obtained favored the proliferation rate of DPSCs and the deposition of calcium phosphate.

## 1. Introduction

In this study, we focused our interest on cortical membranes, commonly used in oral surgical procedures, in order to improve their features thanks to a covalent enrichment with graphene oxide (GO). In particular, we used a type of cortical membrane, namely Osteobiol^®^ Lamina provided by Tecnoss. Laminas, created by a registered trademark of Tecnoss, are made up of cortical bone of heterologous origin and are demonstrated to increase the rate of physiological resorption of the material [1]. Laminas have the compactness of bone tissue as well as a flexibility and adaptability that derives from the superficial decalcification process tuned for their preparation [1]. These materials are therefore used to improve bone tissue regeneration in cases in which it is important to reserve a space [2]. It is important to note that these tissue-derived materials are generally brittle and characterized by a low resistance to fracture. These drawbacks were overcome by enriching the original material, i.e., hydroxyapatite, with different additives such as alginate/chitosan [3], titania [4] or carbon nanotubes (CNT) [5].

Nowadays, graphene has emerged as a great alternative material for applications in biomedical and regenerative engineering. Graphene is a two-dimensional (2D) carbon-based material which has *sp^2^* bonded carbon atoms arranged in a honeycomb lattice structure, with extraordinary electrical, physical, and optical properties [6]. Since its discovery, graphene and its derivatives have been widely investigated for the development of electrical devices and for biomedical applications such as drug delivery systems, biosensors, and regenerative therapies [7]. Mechanically, graphene, despite its flexibility, appears to be one of the strongest materials ever tested. [6] It is transparent, able to conduct electricity and heat better than metals [8], chemically inert, and stable [9]. An increasing number of studies have recently focused on the expansion of new potential applications of graphene nanomaterials, with the aim to highlight the benefits of their use and to improve the application of these nanomaterials [10].

Despite these properties, graphene has a very low solubility in both organic and aqueous solvents. For this reason, hydrophilic graphene derivatives, namely Graphene Oxide (GO), have been widely used and tested for pharmaceutical and biomedical applications. GO is hydrophilic, does not tend to form aggregates, and is highly and homogeneously dispersible in water. GO has been demonstrated to be a biocompatible material whose limited cytotoxicity depends on final concentration, shape, sheet size, dispersibility, and degree of surface functionalization [10]. GO has been investigated for its ability to enhance the proliferation and differentiation of several types of stem cells [11].

The aim of this study was to achieve the covalent functionalization of Laminas, by exploiting, via a simple, cheap, and effective protocol, the capacity of oxygenated groups of GO to interact with cortical membrane surfaces. Indeed, previously investigated GO-coatings [12,13,14] were obtained by simply depositing GO on the elected substrates and therefore exploiting weak London, Van der Waals, or hydrogen-bonding interactions. The concentrations of GO chosen are those that, in preliminary biological assays and in previous studies [13,14], demonstrated not to be toxic for fibroblast cells and favor osteogenic differentiation in dental pulp stem cells (DPSCs) on collagen membranes. The idea is to demonstrate the ability of graphene oxide to improve Laminas biological properties as well as promote the adhesion, the growth and the osteogenic differentiation of DPSCs (Dental Pulp Stem Cells). DPSCs were chosen because of the easy access to the site collection. Besides, DPSCs have an extensive differentiation ability and their capacity to interact with biomaterials makes them ideal for tissue reconstruction [11].

## 2. Materials and Methods

### 2.1. Materials

Cortical membranes (0.5 × 0.5 × 0.2 cm) (Ostebiol^®^ Lamina, Tecnoss) were a gift of Tecnoss dental s.r.l. Pianezza (TO), Italy. GO was purchased from Graphenea, San Sebastian, Spain as an aqueous solution of 4 mg/mL GO. This solution was diluted at the elected concentration and bath ultrasonicated for 10 min (Elmasonic P60H, 37 kHz, 180 W) before use.

All other reagents were product of analytical grade from Merck KGaA, Darmstadt, Germany and they were used as received.

### 2.2. Enrichment with Graphene Oxide

In order to prepare GO-coated Laminas, a protocol of covalent functionalization was optimized. Firstly, the cortical membranes was activated by using a UV/ozone lamp (PSD-UV4 Novascan UV Ozone System Base model, Novascan Technologies, Boone, NC, USA) for 15 min on each side. This permits the subsequent coating with the functional groups. Secondly, the Laminas were dipped in 1 M ethanolic solution of 3-aminopropyl triethoxysilane (APTES, commercial sample from Merck KGaA, Darmstadt, Germany) for 3 h to obtain a thin, stable aminosilane layer on the activated membranes. The so obtained aminosilane-functionalized membranes were rinsed with ethanol and deionized water. Thirdly, these aminosilane-functionalized cortical membranes were dipped in graphene oxide aqueous solution of two different concentrations, 5 or 10 µg/mL. In particular, 4 mL of homogenous dispersion of GO in water were added to 10 cortical membranes (ca. 21 mg) in a baker. The GO solution was left into contact with samples overnight. Finally, membranes were left to dry at room temperature.

Samples were transferred in a 48 multi-well plate for the in vitro tests.

### 2.3. Sterilization of Cortical Membranes

Both pure and GO-coated Laminas were irradiated by using UV irradiation in the Herasafe KS 15, class II, type A2 biological safe cabinet (Thermo Fisher Scientific, North Logan, UT, USA) for 1 h on each side in order to sterilize the specimens.

### 2.4. Apparatus for Chemico-Physical Characterization of Laminas

Thermo-gravimetric analyses (TGA) were recorded on a TGA Q500 (TA Instruments, New Castle, DE, USA) on ca. 12 mg sample. The runs were performed under nitrogen atmosphere by equilibrating the samples at 100 °C for 20 min, following a ramp at 10 °C/min up to 800 °C.

The morphology of Lamina and GO-coated Laminas was evaluated by Atomic Force Microscopy (AFM), using a Multimode 8 Bruker AFM microscope (Bruker, Milan, Italy) coupled with a Nanoscope V controller and commercial silicon tips (RTESPA 300, resonance frequency of 300 kHz and nominal elastic constant of 40 N·m^−1^) with a typical apex radius of 8 nm in Peak Force and ScanAsyst™ in air mode. 

By using this mode, it was possible, from the height panel, to calculate roughness and, from the force curves recorded at various points, to calculate the Young’s modulus. In particular, NanoScope Analysis software 1.8 enables to select the force curves registered at each point of the scanned surface and, from each force curve, to calculate the Young’s modulus by fitting the linear part of the retracting curve via a hertzian model. The deflection sensitivity and tip radius were calibrated, prior to use, against standard sapphire.

### 2.5. Isolation and Culture of DPSCs

The Local Ethical Committee of the University “G. d’Annunzio” Chieti-Pescara approved the project (approval number 1173, date of approval 31/03/2016), in agreement with the Declaration of Helsinki. Dental pulps were extracted from third molars derived from young male and female people (age range 18–28 years) which underwent surgical procedures. All patients signed informed consent. The study involved only impacted teeth without dental pathologies. After the extraction, the surrounding tissues were mechanically eliminated and processed as reported in our previous work [15].

Samples were rinsed with phosphate-buffered saline (PBS), maintained in Minimum Essential Medium (α-MEM) (Merck KGaA, Darmstadt, Germany) supplemented with 10% of Foetal Bovine Serum (FBS) and 1% antibiotics (penicillin/streptavidin mixture, EuroClone S.p.A, Milan, Italy) and sent to the laboratory for stem cells extraction [15]. When cells covered 80–90% of the flask area (subconfluence condition) they were subcultured. Antigen expression of CD29, CD45, CD105, CD73 CD90 and SSEA-4 was checked by flow cytometry [15].

### 2.6. DPSCs Culture on Laminas

Cells from the fourth passage (Figure S1 of the Supporting Information) were seeded on Laminas, 10,000 cells/cm^2^ were used and cultured up to 28 days. Two hundred twenty Laminas for each experiment were used, fifty-five Laminas were used for each experimental point. Experiments were repeated for three times. At the established times cells were harvested and processed for the required analyses. The cells were cultured in α-MEM medium supplemented with differentiation factors such as 10 nM dexamethasone, 0.2 mM ascorbic acid and 10 mM β-glycerophosphate, as reported elsewhere [16,17].

### 2.7. Scannsion Electron Microscopy (SEM) Analysis

Samples were fixed with 1.25% glutaraldehyde in 0.1 M cacodylate buffer for 30 min, dehydrated through alcohol ascending series and then dried with hexamethyldisilazane followed by gold-coating. All micrographs were obtained at 15 kV on compact desktop Phenom XL SEM microscope.

### 2.8. Alamar Blue Cell Viability Assay

The Alamar blue test was performed in triplicate for each experimental sample at each experimental time. Cells viability was measured after 3, 7, 14 and 28 days of culture. The test is based on the reduction of Alamar blue reagent (Thermo Scientific, Rockford, IL, USA), performed only by viable cells, into a red product. At established experimental times the medium was replaced by a new one added with 10% of Alamar blue reagent, incubated for 4 h at 37 °C. A spectrophotometric reading at 570 and 600 nm wavelength was performed. The negative control was established as the value obtained without cells. The percentage reduction of Alamar blue reagent was calculated following the manufacturer instructions.

### 2.9. Lactate Dehydrogenase (LDH) Cytotoxicity Assay

To evaluate biomaterial cytotoxicity, LDH release into the medium was measured by means of CytoTox 96 non-radioactive cytotoxicity assay (Promega, Madison, WI, USA) at each time point (3, 7, 14 and 28 days). The LDH leakage in each well was normalized to the lysis value obtained in a lysis well of the same experimental point in which a lysis solution was added to the medium 

### 2.10. Alizarin Red S (ARS) Staining

Alizarin red S is a calcium-sensing dye. DPSCs, differentiated towards the osteogenic phenotype, are able to deposit and to induce the mineralization of extracellular matrix rich in calcium phosphate, which can be identified by Alizarin red S. Calcium deposits are detectable for their bright orange-red color.

The DPSCs in each well were rinsed twice with PBS, PBS was discarded and DPSCs were fixed in paraformaldehyde 4% for 15 min at room temperature and then washed with deionized water. Alizarin red S staining solution 40 mM (Merck KGaA, Darmstadt, Germany) was added to each well and probed for 20 min at room temperature (RT) on a shaker. The wells were washed for five times in deionized water. Calcium deposits, stained in orange-red, were dissolved as follows: 10% acetic acid was added under shaking for 30 min. Laminas were scraped, the liquid containing deposits was collected and vortexed in a tube. Previously heated warm mineral oil (Merck KGaA, Darmstadt, Germany) was added, the tube maintained on ice for 5 min and eventually centrifuged at 20,000 *g* for 15 min. The supernatant was discarded and 10% ammonium hydroxide (Merck KGaA, Darmstadt, Germany) was added. The final solution was analyzed by a spectrophotometric reading performed at 405 nm wavelength.

### 2.11. Statistical Analysis

SPSS software version 16.0 (SPSS, Inc., Chicago, IL, USA) (Statistical Package for Social Science) and GraphPad Prism 5 were used to perform statistical analysis. Data were evaluated using one-way analysis of variance followed by the Tukey-Kramer post-hoc test. The results were expressed as the mean ± standard deviation (SD). *P* < 0.05 was considered to indicate a statistically significant difference.

## 3. Results

**Figure 1:**
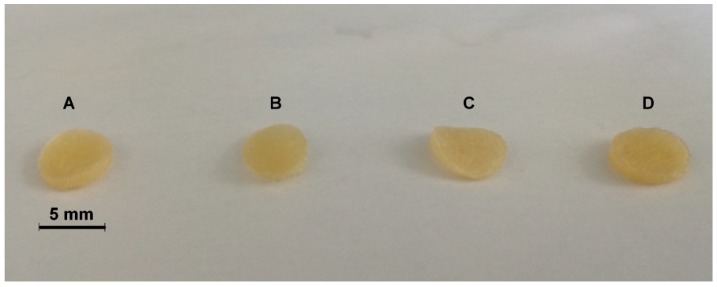
Photographs of (**A**) pure Lamina, (**B**) Lamina functionalized with 3-aminopropyl triethoxysilane (APTES) (see Experimental Section 2.2), (**C**) Lamina enriched with 5 µg/mL graphene oxide (GO) and (**D**) Lamina enriched with 10 µg/mL GO.

Laminas were enriched with GO at two different concentrations, 5 and 10 µg/mL. Photos of the obtained enriched cortical membranes are reported in Figure 1.

**Figure 2:**
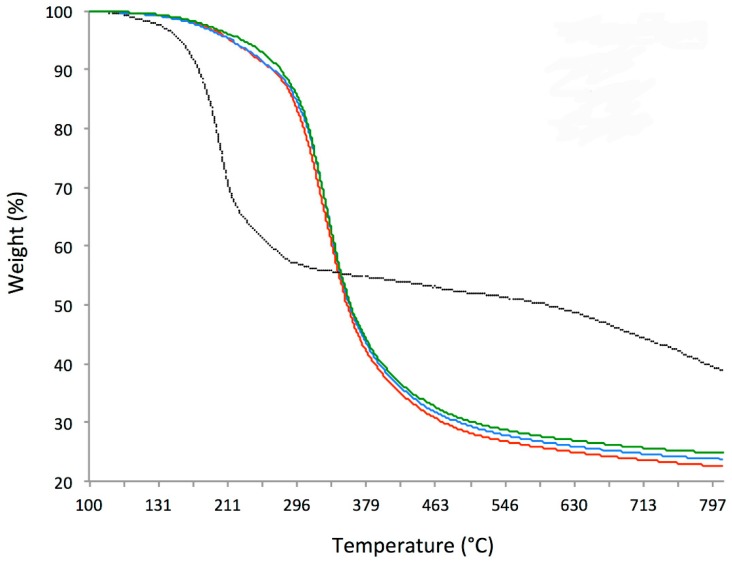
Thermo-gravimetric analyses (TGA) of graphene oxide (black curve), bare Lamina (red curve), 5 µg/mL GO enriched Lamina (blue curve) and Lamina enriched with 10 µg/mL GO (green curve).

We tried to evaluate the amount of GO covalently attached to the functionalized Lamina by using TGA (Figure 2). While the GO sample presents the common behavior with an important weight loss at 200 °C, the Laminas profiles show a consistent weight loss at around 325 °C as for 5 µg/mL GO and 10 µg/mL GO (330 °C), even though for the last preparation a small implement of stability can be appreciated up to 270 °C with a difference in weight loss of 1.3% (9.1% vs. 10.4% in the case of control and 5 µg/mL GO).

**Figure 3:**
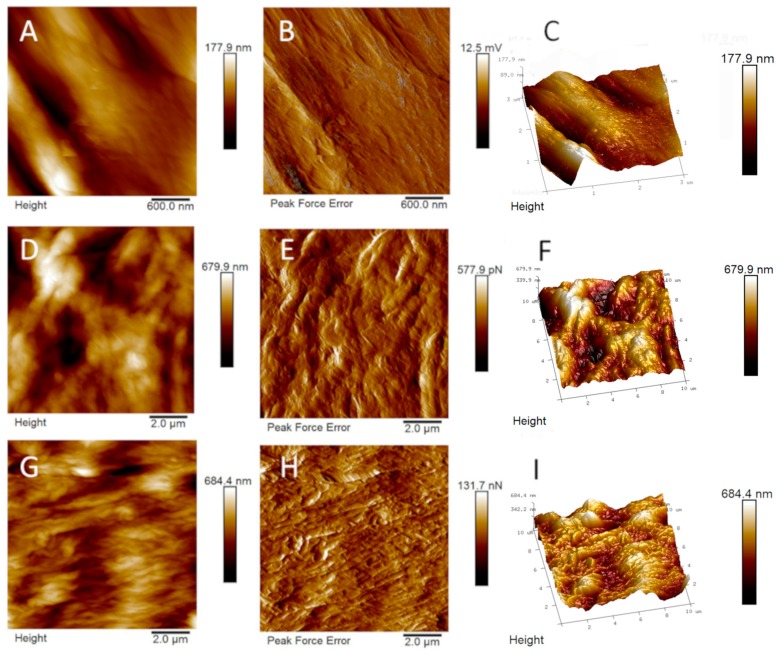
(**A**,**D**,**G**) Topographical, (**B**,**E**,**H**) Peak force error and (**C**,**F**,**I**) Three-dimensional Atomic Force Miscroscopy (AFM) images of bare Lamina (upper line), Lamina enriched with 5 µg/mL of GO (central line) and Lamina enriched with 10 µg/mL GO (bottom line).

Bare and GO-enriched cortical membranes were analyzed by using AFM. In Figure 3, topographical and tridimensional micrographs as well as peak force error images of bare and GO-enriched (5 µg/mL and 10 µg/mL) cortical membranes are reported. From the images reported the changes of the topography of the surface on enrichment with GO are evident.

From the height panel the Nanoscope analysis 1.8 software (Bruker, Milan, Itay) is able to recover the roughness indexes (i.e., the root-mean square roughness, Rq; the mean absolute value of the surface high deviations, Ra; i.e., the distance between the highest and lowest data points in the image, Rmax; the root-mean square of the surface slope, Sdq, and the ratio between the developed and the planar area, Sdr). We calculated these indexes for the bare membrane as the mean values of roughness recovered from two panels with a total surface area of 18 µm^2^, were Rq = 53.0 ± 10.2 nm, Ra = 43.3 ± 9.3 nm, Rmax 255.5 ± 20.5 nm, Sdq 12.7 ± 1.6°, and Sdr 2.5 ± 0.5%. The roughness indexes, calculated as the mean values recovered from three panels with a total surface area of 300 µm^2^, were Rq = 216.0 ± 21.2 nm, Ra = 175.0 ± 21.9 nm, Rmax 1303.3 ± 96.3 nm, Sdq 21.4 ± 1.8°, and Sdr 7.16 ± 1.0% for the GO-coated sample with 5 µg/mL and Rq = 254.7 ± 56.12 nm, Ra = 205.7 ± 46.5 nm, Rmax 1311.0 ± 282.0 nm, Sdq 25.9 ± 10.9°, and Sdr 11.5 ± 7.6% for the GO-coated sample with 10 µg/mL (See Supporting Information, Figures S8–S10).

**Figure 4:**
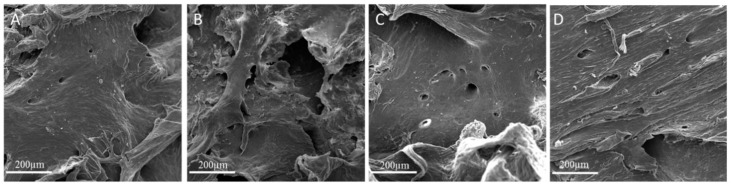
Scansion electron microscopy (SEM) images of (**A**) bare Lamina, (**B**) APTES-treated Lamina, (**C**) 5 µg/mL GO-coated Lamina and (**D**) 10 µg/mL GO-coated Lamina. Magnification 3000×. Scale bar: 200 µm.

Scansion electron microscopy (SEM) experiments (Figure 4) allowed to observe morphology differences in the investigated Laminas. As seen from SEM images (compare Figure 4B and Figure 4A) the covalent functionalization with amino silane brought about a significant deformation of Lamina Surface. The subsequent coating with GO restored the typical layered structure of GO [13], with layered regions increasing on increasing GO concentration (compare Figure 4C and Figure 4D).

DPSCs were cultured on Laminas with medium containing differentiation factors up to 28 days; 3, 7, 14, and 21 days were chosen as experimental times.

**Figure 5:**
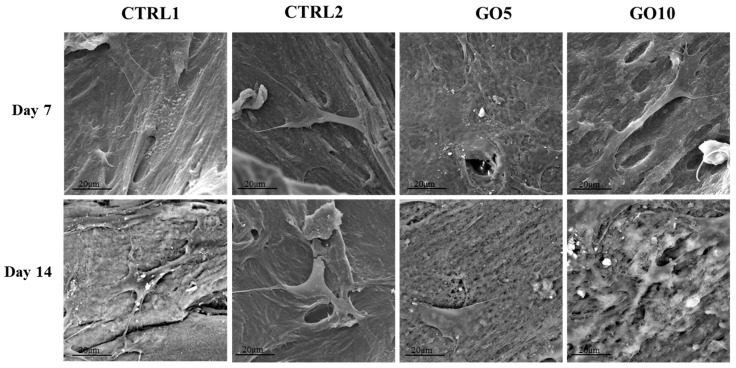
SEM images of Dental Pulp Stem Cells (DPSC)) cultured on bare Laminas (CTRL1), APTES-treated (CTRL2), 5 µg/mL GO-coated (GO5) and 10 µg/mL GO-coated (GO10) Laminas for 7 and 14 days. Magnification 3000×.

Before starting the evaluation of the biological parameters, an SEM analysis, after 7 and 14 days of culture, was performed in order to evaluate DPSCs morphology, spread and adhesion on Laminas. After 7 days of culture, cells are detectable on all the observed experimental points: DPSCs cultured on control Laminas are flat and spread throughout the surface, some granules of inorganic matrix are starting to be deposited. DPSCs cultured on APTES-treated Laminas appear isolated, with short cytoplasmic extensions, probably suffering and they do not cover all the surface of the Lamina. DPSCs grown on both 5 µg/mL GO- and 10 µg/mL GO-coated Laminas form a uniform cell layer on the biomaterial, they appear completely flat and in close contact with each other; an isolated cell is not recognizable. White granules of inorganic matrix can be identified especially on 5 µg/mL GO-coated Lamina. The same trend is revealed after 14 days of culture (Figure 5). 

**Figure 6:**
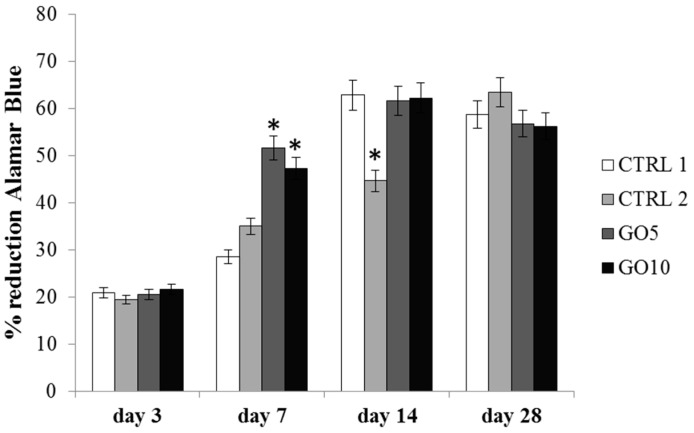
Alamar blue assay in DPSC cultured on bare Laminas (CTRL1), APTES-treated (CTRL2), 5 µg/mL GO-coated (GO5) and 10 µg/mL GO-coated (GO10) Laminas for 3, 7, 14, and 28 days. Forty Laminas were used for each experimental point, ten Laminas per experimental time. The histogram represents Alamar blue reduction percentage, data shown are the mean (±SD) of three separate experiments. Zero time % reduction Alamar Blue: 15.72%; * Day 7: GO5 and GO10 Laminas vs. control (CTRL1) Laminas *p* < 0.001; Day 14 control Laminas, GO5-coated and GO-10 coated Laminas vs. APTES-treated Laminas (CTRL2) *p* < 0.001.

Cell viability was measured by means of Alamar Blue Assay after 3, 7, 14, and 28 days. After 3 days of culture the viability level does not show any significant difference among the tested experimental points, whereas after 7 days of culture an appreciable increase in viability level is recordable when DPSCs are cultured on GO-enriched Laminas. In particular, the cell viability is almost doubled for DPSCs cultured on 5 µg/mL GO-coated Laminas with respect to the control and it is comparably high for DPSCs cultured on 10 µg/mL GO-coated Laminas. Both the percentage of Alamar Blue reduction recorded on 5 µg/mL GO- and 10 µg/mL GO-coated membranes are statistically significant with respect to the control (*p* < 0.001). The metabolic activity of cell cultured on control and on GO-coated Laminas further augments (Figure 6) until 14 days of culture. By day 14, the number of viable cells reach a plateau, suggesting that those surfaces are advancing into confluence. On the other hand, it is worth noting that the proliferation rate of DPSCs cultured on APTES-treated Laminas is much lower, reaching the maximum percentage of Alamar Blue reduction at 28 days, when the difference with the other samples cancels out.

**Figure 7:**
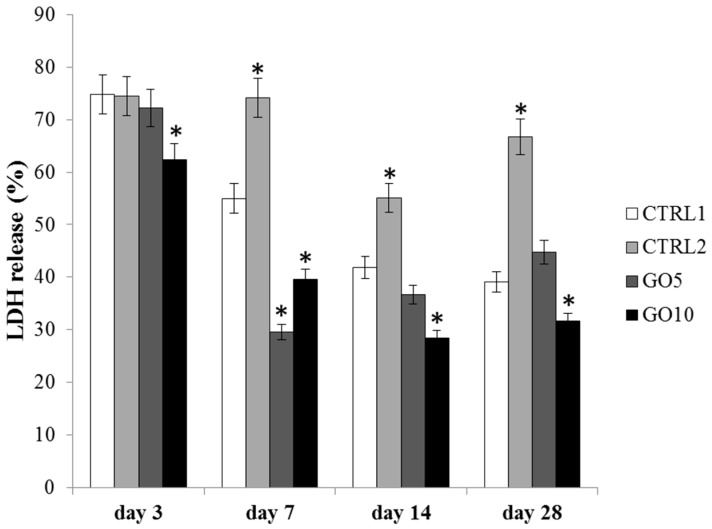
Lactate Dehydrogenase (LDH) assay of DPSC cultured on bare Laminas (CTRL1), APTES-treated (CTRL2), 5 µg/mL GO-coated (GO5) and 10 µg/mL GO-coated (GO10) Laminas for 3, 7, 14, and 28 days. Forty Laminas were used for each experimental point, ten Laminas per experimental time. Released LDH is reported as percentage. Data shown are the mean (±SD) of three separate experiments. Zero time LDH release (%): 73.06 * Day 3: 10 µg/mL GO-coated Laminas (GO10) vs. control (CTRL1) *p* < 0.05; * Day 7: APTES-treated, 5 µg/mL GO-coated (GO5) and 10 µg/mL GO-coated Laminas (GO10) vs. control (CTRL1) *p* < 0.001; control Laminas, APTES-treated Laminas vs. 5 µg/mL GO-coated Laminas (GO5) *p* < 0.001; control Laminas, APTES-treated Laminas vs. 10 µg/mL GO-coated Laminas (GO10) *p* < 0.001; * Day 14, day 28: APTES-treated, GO10 Laminas vs. control (CTRL1) Laminas *p* < 0.005.

The cytotoxicity of the biomaterial was evaluated through LDH assay by measuring the percentage of released LDH within the culture medium after 3, 7, 14, and 28 days of culture. After 3 days of culture the cytotoxicity level is higher than 70% for all tested samples except for DPSCs cultured on 10 µg/mL GO-coated Laminas. In fact, this sample shows a released LDH percentage significantly lower than that measured for the three others samples. After 7 days of culture the cytotoxicity level starts to decrease with respect to that measured after 3 days of culture in all the investigated samples except for DPSCs cultured on APTES-treated Laminas. In fact, the released LDH percentage for this sample appears still higher than 70%, whereas the percentage decreases under 60% for cells grown on control Laminas and under 40% for DPSCs cultured on GO-coated Laminas. The cytotoxicity level does not change thereafter for GO-coated samples and a statistically significant reduction of released LDH percentage is detected for DPSCs grown on 10 µg/mL GO-coated Laminas with respect to the control (Figure 7). Again DPSCs cultured on APTES-treated Laminas show the highest released LDH percentage (>50%) among the four Laminas investigated.

**Figure 8:**
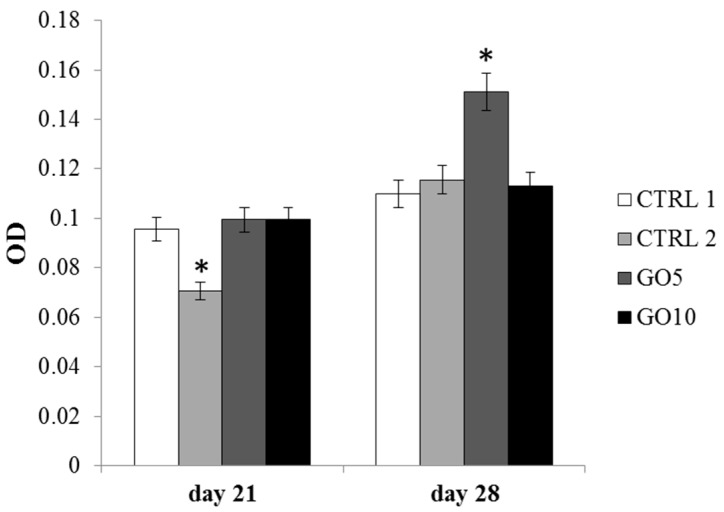
The histogram represents optical density (OD) values of solubilized calcium deposits (orange-red stained) obtained after Alizarin Red staining on bare Laminas (CTRL1), APTES-treated (CTRL2), 5 µg/mL GO-coated (GO5) and 10 µg/mL GO-coated (GO10) Laminas. Twenty Laminas were used for each experimental point, ten Laminas per experimental time. Data shown are the mean (±SD) of three separate experiments. * Day 21: APTES-treated Laminas vs. control (CTRL1) Laminas *p* < 0.005; * Day 28: control Laminas, APTES-treated, 10 µg/mL GO-coated Laminas (GO10) vs. 5 µg/mL GO Laminas (GO5) *p* < 0.005.

Bone matrix deposition was measured through Alizarin Red staining, a calcium-sensing dye able to identify extracellular quantities of calcium phosphate. Alizarin Red staining was performed after 21 and 28 days of culture. After 21 days of culture, a marked decrease of synthetized calcium phosphate could be detected in DPSCs cultured on APTES-treated Laminas compared with the control and with GO-coated Laminas. Conversely, after 28 days of culture, a statistically significant increase in calcium phosphate deposition is detected in DPSCs cultured on 5 µg/mL GO-enriched Laminas with respect to all other tested samples (Figure 8).

## 4. Discussion

Laminas were covalently enriched with GO, by using APTES as the linker between the Lamina and the GO sheets. This type of functionalization was chosen in order to create on the biomaterial a layer of graphene oxide covalently bound to the scaffold. As a matter of fact, in a previous study [12], porcine bone granules, enriched with GO by exploiting simple physical deposition, were implanted in animals for three months and excess GO was detected in the form of GO aggregates in both hard and soft tissues. 

TGA did not allow us to properly quantify the amount of GO functionalized onto Laminas, because the amount of GO was very low and the two materials, cortical membrane and GO, evidenced a weight loss at similar temperatures. An approximately 1.3% GO could be calculated at least for the more concentrated 10 µg/mL sample.

Nevertheless, the GO demonstrated good distribution, through AFM and SEM analyses, on the Lamina and changed completely the appearance of the surface of the cortical membrane. Despite SEM appearance, the enrichment with GO rendered the surface more rough, as already recently evidenced in the case of GO-enrichment of porcine bone granules and collagen membranes [12,13]. Indeed, SEM is not the best technique in order to discriminate GO coverage percentage, but SEM images highlight the formation of layered GO over the Lamina surface (Figure 4). AFM measurements instead evidenced that GO-enriched Laminas are rougher than bare membranes and present a much wrinkled structure (compare panels B, E, and H and C, F, and I in Figure 3).

In particular, the calculated roughness indexes, Rq and Ra, indicate that the non-coated membrane is characterized by lower peaks and therefore a lower roughness as compared to the GO-coated samples, as confirmed by Rmax values. The surface indexes, Sdr and Sdq, confirmed Ra, Rq, and Rmax data because they evidenced a surface enlargement induced by the presence of GO with more and steep peaks, respectively. Nevertheless no significant differences were highlighted between the samples enriched with 5 or 10 µg/mL GO, likely due to a saturation-like effect of the surface with the lowest concentration of GO investigated.

The measured Young’s elastic modulus, obtained as an average value calculated from 20–25 force curves in samples of 10 µm × 10 µm dimensions, is 0.77 ± 0.46, 0.83 ± 0.62, and 1.00 ± 0.27 GPa in bare cortical membrane, 5 µg/mL and 10 µg/mL GO-coated membranes, respectively (See Supporting Information, Figures S2–S7). Therefore it does not change very much on GO enrichment, although a small increase could be monitored on increasing the concentration of GO, indicating that GO contributes to matrix stiffening. It is interesting to note that the SD is very high (60%) in the commercial cortical membrane due to the presence of pores and defects, keeps a high value for 5 µg/mL coated Laminas but reduces in Laminas enriched with 10 µg/mL GO, thus highlighting the presence of a homogeneous GO layer in the latter sample. These values are of the same order of those of polyethylene (1.5–2 GPa) and polystyrene (3–3.5 GPa), substrates which have been previously demonstrated to be ideal substrates for the growth of stem cells. 

By considering that mesenchymal stem cells demonstrated [18] to sense matrix elasticity and preferentially differentiate depending of the stiffness of the substrate, such values, indicative of stiff matrices, appear proper to favor expression of an osteogenic lineage.

Laminas coated with 5 µg/mL and 10 µg/mL GO were then tested in an in vitro model, by seeding and culturing DPSCs on Laminas surface, in order to evaluate the biocompatibility of GO-enriched Laminas, in terms of cell viability, cytotoxicity, and mineralized matrix deposition. 

During the DSPCs differentiation, GO enrichment positively modifies the biological parameters evaluated, thus indicating a good tolerability and an improved biocompatibility. Indeed, GO enrichment, both at 5 and 10 µg/mL concentration, improves the cell spread throughout the surface of the biomaterial thus allowing to hypothesize that GO enrichment is able to promote the adhesion process and favor the formation of a uniform cell layer (See Figure 5). Moreover, GO coating enhanced the growth rate of DPSCs. In fact, cells seeded on GO-coated Laminas after 7 days of culture show cell viability values two-fold that of bare Laminas evidencing confluence after 14 days of culture (see Figure 6). It is worth noting that the above mentioned GO-induced cell viability value is not related to GO concentration as both samples evidence the same effect. Nevertheless it is important to stress that these similarities can be explained by the above mentioned saturation-like effect, with GO covering almost completely the Lamina surface already at the lowest investigated concentration. These results may be associated to the capacity of GO to favor protein adsorption [19]. Indeed, it has been demonstrated that serum proteins absorb quickly and spontaneously to graphene oxide surface to form a corona complex [20] and this adsorption, that demonstrate to be selective for different proteins, may affect adhesion, proliferation, and/or osteogenic differentiation of stem cells [19]. It was also demonstrated that induction of human mesenchymal stem cells (hMSC) differentiation towards different tissue lineages depended on the degree of π-π stacking with graphene and hydrogen bonding as well as electrostatic interactions with GO [19]. In the present case, a positive effect towards adhesion and viability of DPSCs may be induced also by the highly wrinkled surface associated to a small increase in stiffness obtained on GO enrichment. A similar evidence has been already demonstrated for highly convoluted methacrylate-functionalized GO membrane [21] that favored spontaneous stem cell differentiation towards bone lineage even in the absence of osteogenic growth factors.

The cytotoxicity level also appeared significantly reduced for GO-enriched Laminas during 28 days of culture and actually a statistical significant increase of membrane integrity was detected for Laminas enriched with 10 µg/mL GO at all the investigated times. It is worth noting that a slightly different behavior characterizes 5 µg/mL GO-coated Laminas, with cytotoxicity slightly increasing and reaching that of the bare Laminas at 14 and 28 days of culture. These results show that, although GO functionalization demonstrated to promote favorable biological effects on DSPCs, it is necessary to carefully tune the concentration of GO bound to Laminas in order to reach the best compromise of effectiveness and biocompatibility. Indeed, different studies evidenced that doses, as well as size, is a fundamental parameter to consider in order to fully characterize GO toxicity [22]. On the other hand, a relatively high toxicity was detected for APTES-treated Laminas. Despite an APTES-treated different material, such as nanoparticles, did not show any toxic effect on cell membrane integrity [23], polyamines demonstrated [24] to promote leakage of liposomal content from 1-palmitoyl-2-oleoyl-sn-glycero-3-phosphocholine (POPC) liposomes due to interactions of primary ammonium groups with phospholipidic head groups. Similarly APTES-treated Laminas could promote analogous ammonium-phospholipidic headgroups interactions, thus explaining the sustained LDH leakage from cell membrane and the chronic cytotoxicity responsible for the lower proliferation rate as compared to cells seeded on the other investigated samples. These data are very interesting because they highlight that, after aminosilane-functionalization, the subsequent treatment with GO allows to override the negative effect evidenced in the presence of APTES-treated Laminas on cell cytotoxicity.

These results are further supported by mineralized bone matrix deposition which appears increased after 28 days of culture and therefore at the end of the osteogenic differentiation [25] but only for DPSCs cultured on 5 µg/mL GO-coated Laminas. They highlight the important role of GO as responsible for a faster and more intense promotion of bone matrix deposition. To conclude, this is a preliminary study on biocompatibility and lack of cytotoxicity of the GO-functionalized Laminas and further investigations are needed in order to fully characterize their biological properties. First of all a detailed investigation focused on the tuning of GO concentration needed for the covalent functionalization of Laminas will allow to optimize the risk-to-benefit balance and clarify all the factors affected by GO enrichment.

## 5. Conclusions

This study demonstrated that the relatively homogeneous coating of investigated commercial cortical membranes with GO was relatively easy to obtain. It favored the proliferation rate of DPSCs probably due to the capacity of GO to adsorb proteins present in the medium. Clear evidences of reduced toxicity were evidenced and Laminas enriched with GO 5 µg/mL demonstrated a statistical significant increase of calcium phosphate deposition. The present study is particularly promising and we believe that this material holds potential as useful substrate to facilitate in vivo bone regeneration. Nevertheless it highlights the need to further investigate GO-coated samples in order to tune the concentration of GO that demonstrates the best osteogenic activity and biocompatibility.

## Supplementary Materials

The following are available online at https://www.mdpi.com/2079-4991/9/4/604/s1, Figure S1: DPSCs observed with a light microscope before detachment and seeding for osteoblastic differentiation, Figures S2–S7: Original AFM micrographs and representative force curves of pure Lamina, Lamina enriched with 5 µg/mL GO and 10 µg/mL GO used for the mechanical studies, Figures S8–S10: Original AFM micrographs used for the roughness index calculation of pure Lamina, Lamina enriched with 5 µg/mL GO and 10 µg/mL GO, Tables S1–S3: Roughness indexes for pure Lamina, Lamina enriched with 5 µg/mL GO and 10 µg/mL GO.
