# Supplementary material for: Covalent Decoration of Cortical Membranes with Graphene Oxide as a Substrate for Dental Pulp Stem Cells
Source: Nanomaterials (Basel). 2019 Apr 12;9(4):604. doi: 10.3390/nano9040604 (PMC6523176; doi:10.3390/nano9040604)
Supplement: Supplementary file 1 [file nanomaterials-09-00604-s001.pdf]

Covalent decoration of cortical membranes with graphene oxide as a substrate for Dental Pulp Stem cells

Roberta Di Carlo [1,†] Susi Zara [1,†], Alessia Ventrella [1,†], Gabriella Siani [1], Tatiana Da Ros [2], Giovanna Iezzi [3], Amelia Cataldi [1] and Antonella Fontana [1,*]

[1] Department of Pharmacy, Universy "G. d'Annunzio", Via dei Vestini, 66100 Chieti, Italy
[2] Department of Chemical and Pharmaceutical Sciences, University of Trieste. Piazzale Europa 1, 34127 Trieste, Italy
[3] Department of Medical, Oral and Biotechnological Sciences, Universy "G. d'Annunzio", Via dei Vestini, 66100 Chieti, Italy

* Correspondence: fontana@unich.it; Tel.: +39 0871 3554790
† These Authors contributed equally.

SUPPORTING INFORMATION

S1. Morphology of DSPCs

S2. Original AFM data for the mechanical studies

S1. Morphology of DSPCs

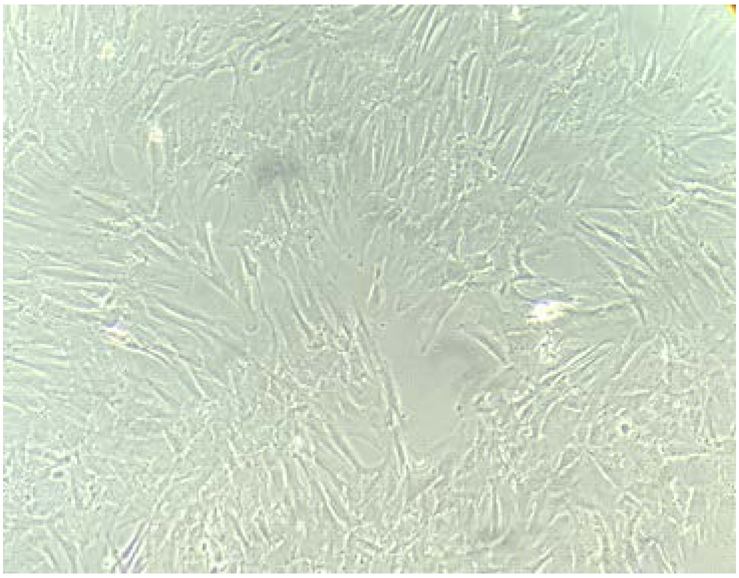

Figure S1. DPSCs observed with a light microscope before detachment and seeding for osteoblastic differentiation. Magnification 10×.

S2. Original AFM data used for the mechanical studies

S2.1 Original AFM micrograph of pure Lamina used for the mechanical studies

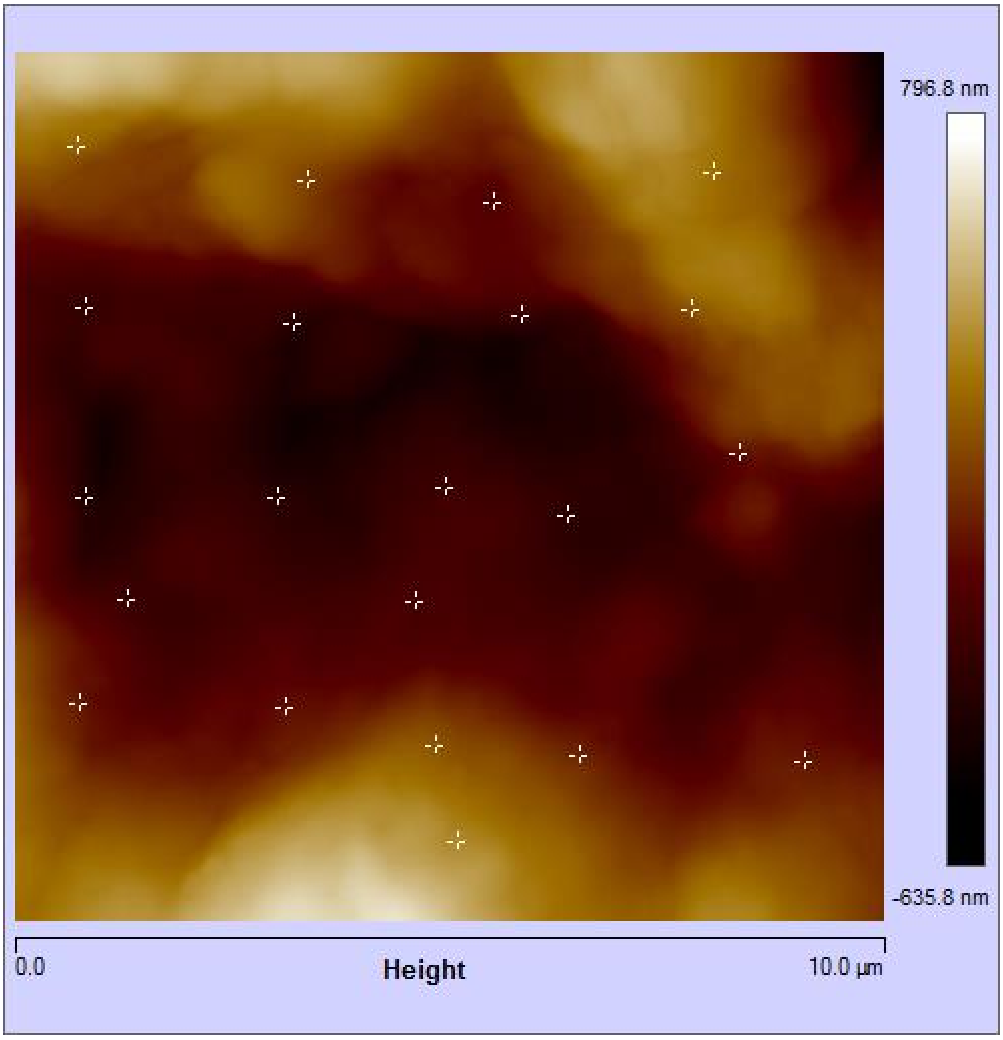

Figure S2. AFM micrograph of topographical height of pure Lamina indicating the points where the force curve has been extracted and used for the calculation of the Young's Modulus.

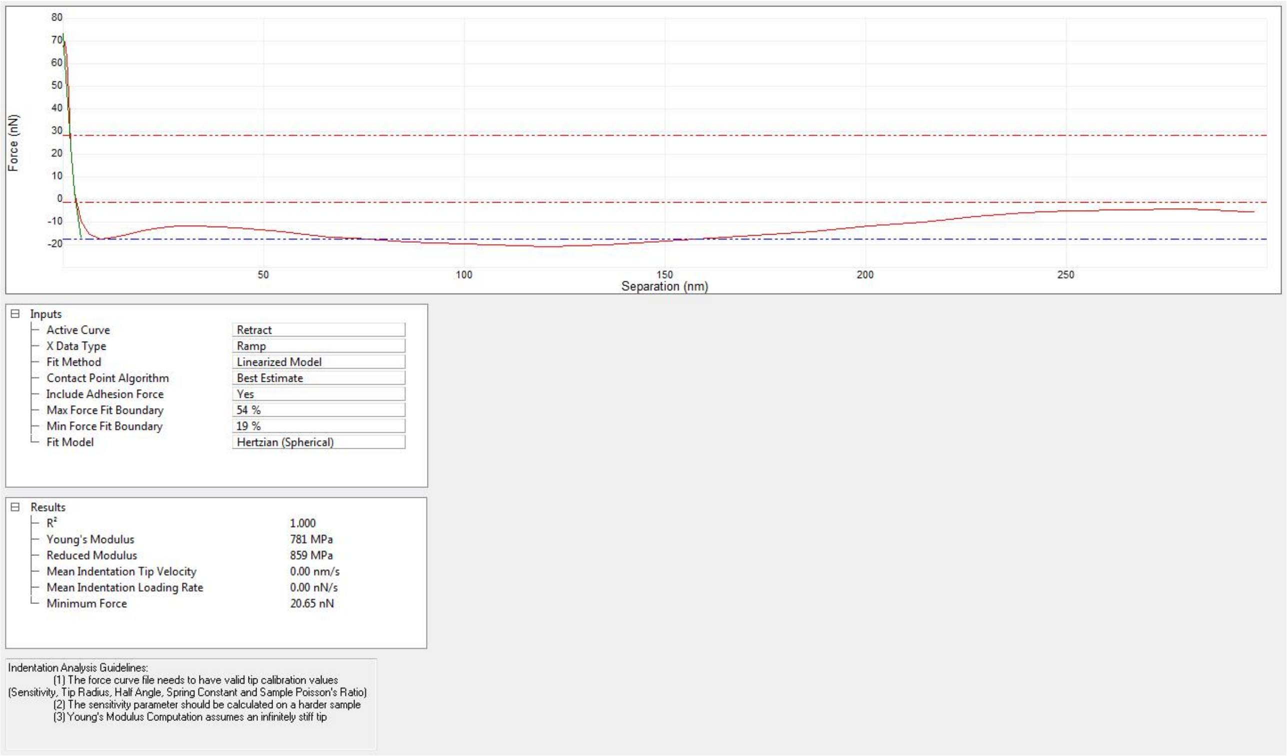

Figure S3. Representative Force Curve and corresponding Young's modulus calculation for pure Lamina. The final value is the average value among 21 data.

S2.2 Original AFM micrograph of Lamina enriched with 5 µg/mL GO used for the mechanical studies

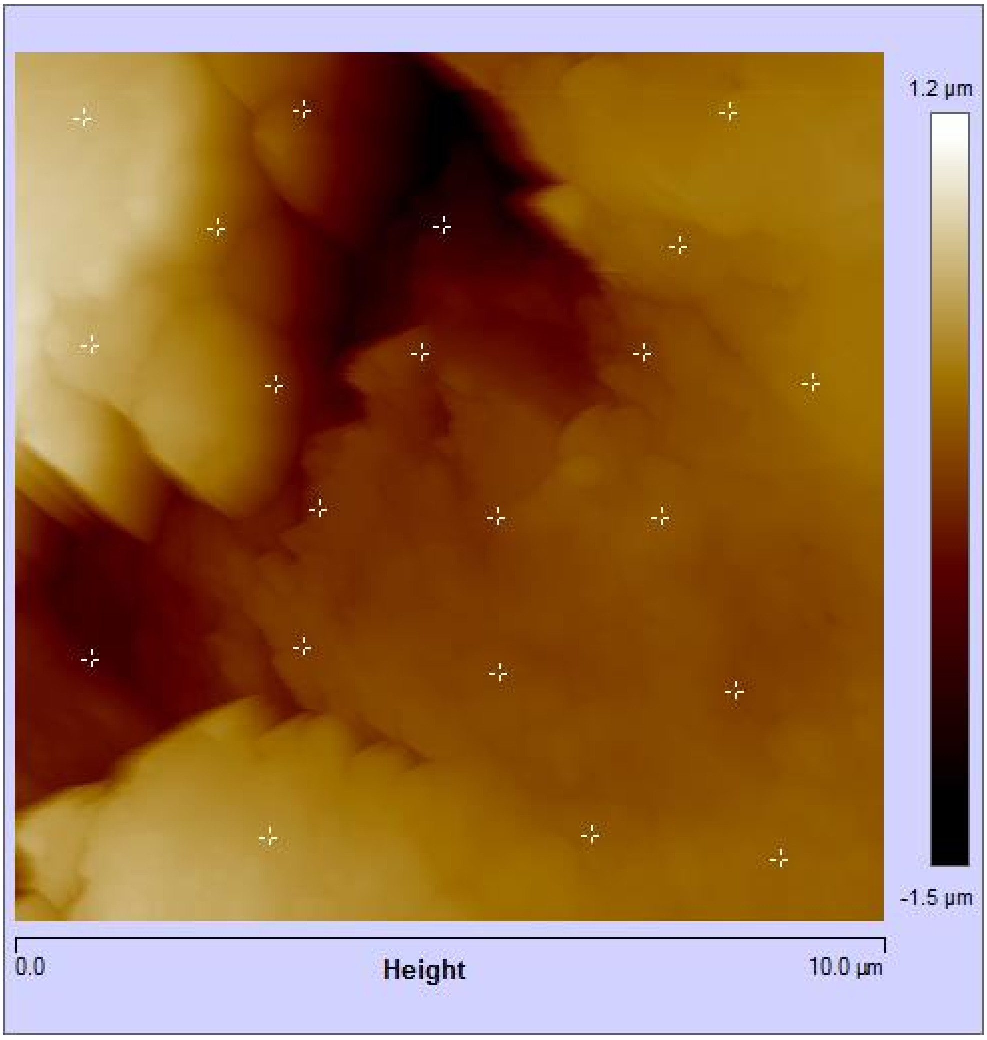

Figure S4. AFM micrograph of topographical height of Lamina enriched with 5 µg/mL GO indicating the points where the force curve has been extracted and used for the calculation of the Young's Modulus.

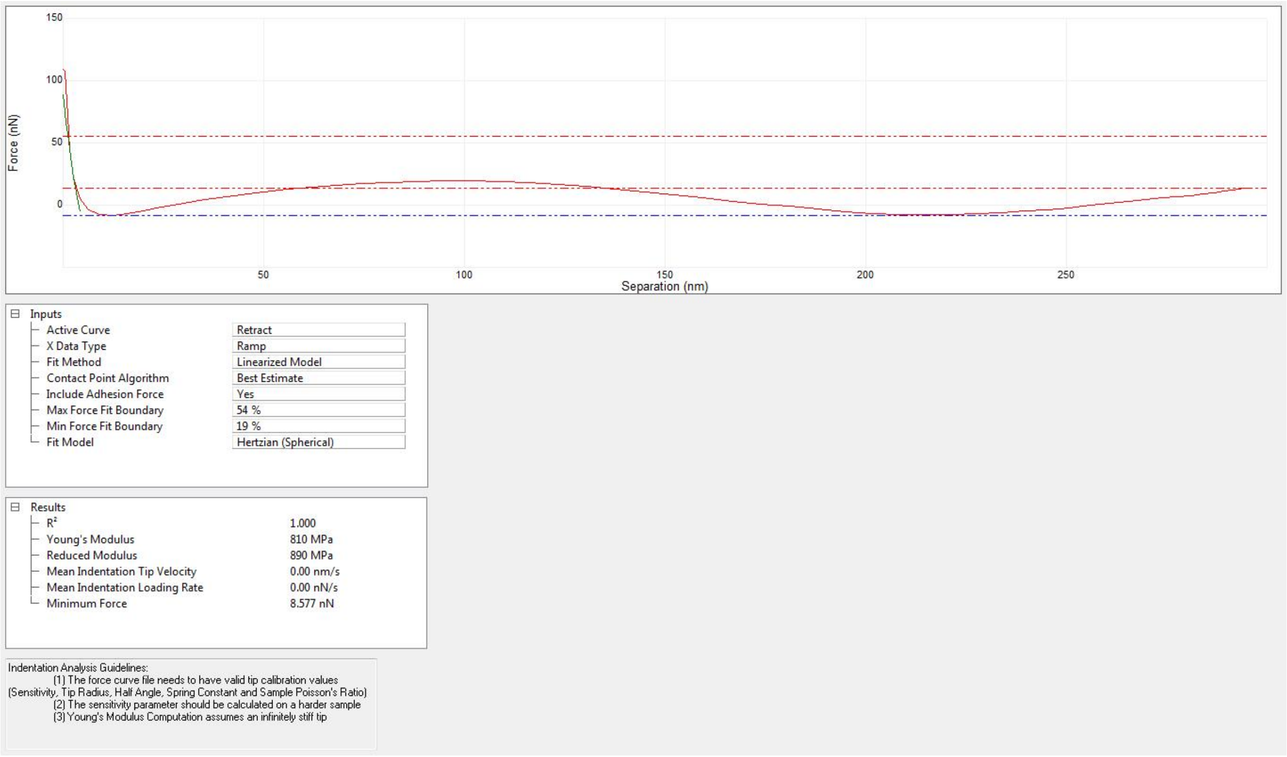

Figure S5. Representative Force Curve and corresponding Young's modulus calculation for Lamina enriched with 5 μg/mL GO. The final value is the average value among 21 data.

S2.3 Original AFM micrograph of Lamina enriched with 10 μg/mL GO used for the mechanical studies

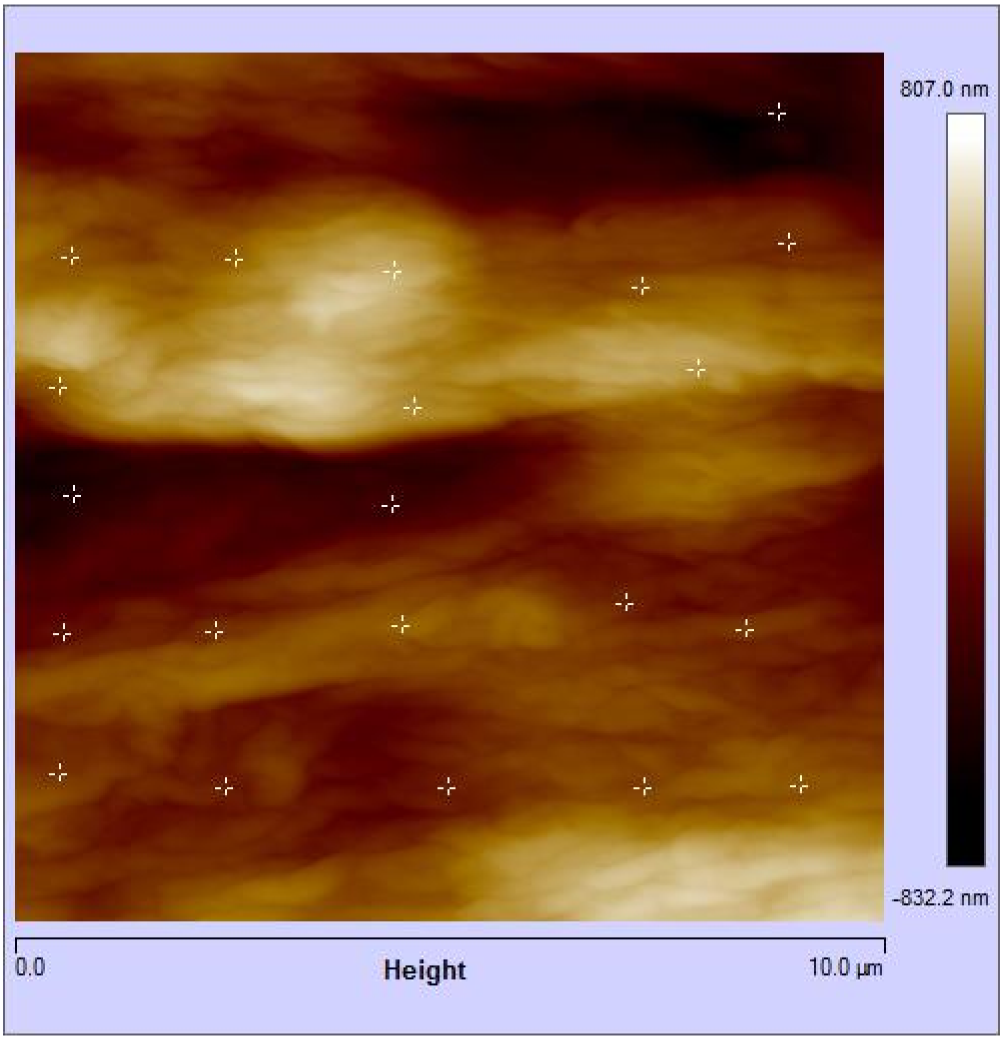

Figure S6. AFM micrograph of topographical height of Lamina enriched with 10 μg/mL GO indicating the points where the force curve has been extracted and used for the calculation of the Young's Modulus.

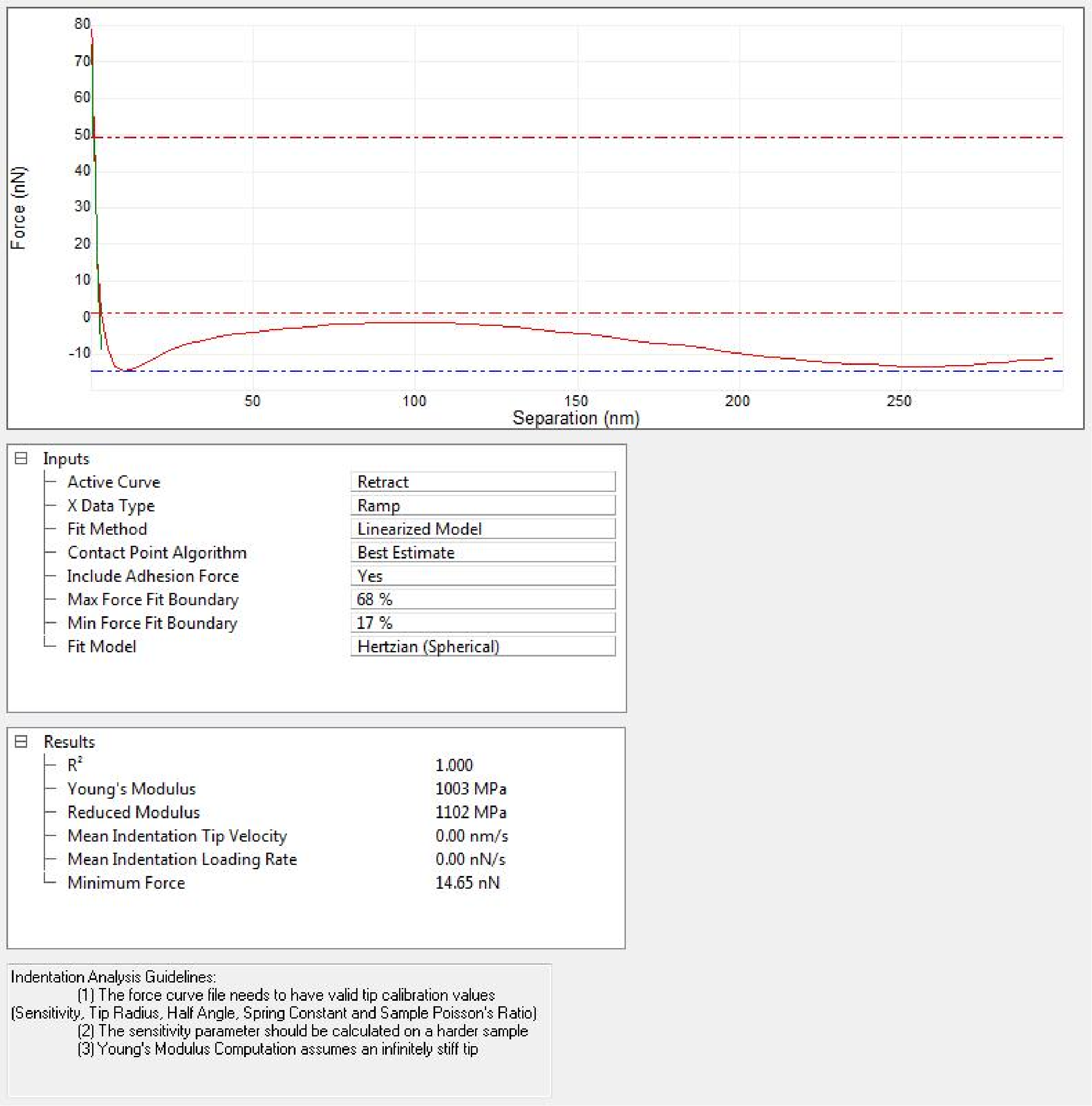

Figure S7. Representative Force Curve and corresponding Young's modulus calculation for Lamina enriched with 10 µg/mL GO. The final value is the average value among 22 data.

S2.4 Original AFM micrographs used for the roughness index calculation

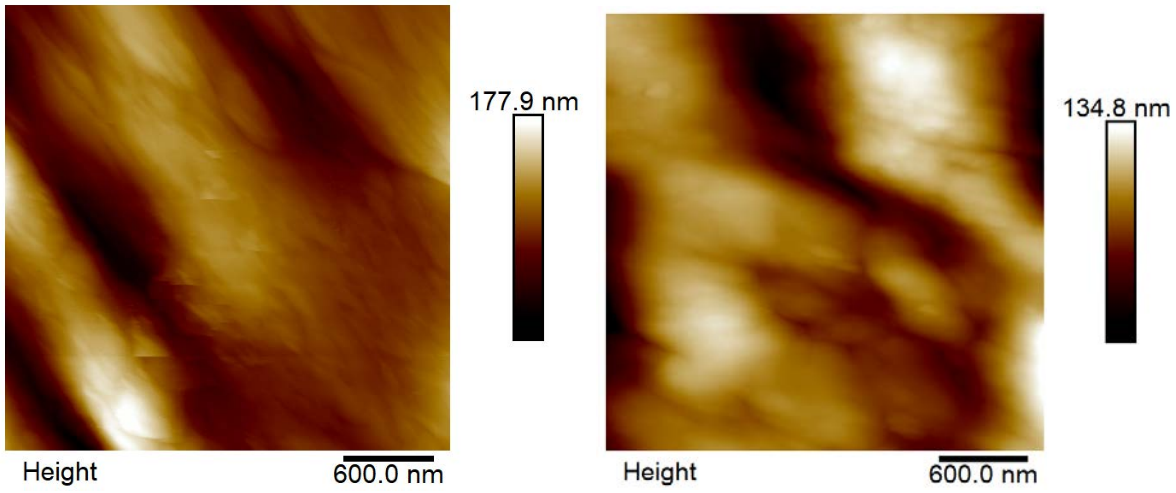

Figure S8. Height panels of bare Lamina sample used for the calculation of roughness indexes.

Table S1. Roughness indexes recovered from Panels of Figure S8 for bare Lamina sample.

Roughness recovered	Ra	Rq	Rmax	Sdq	Sdr
Panel 2	36.7 nm	45.8 nm	241 nm	13.8 °	2.86 %
Panel 2	49.9 nm	60.3 nm	270 nm	11.6 °	2.09 %

S2.5 Original AFM micrographs of Lamina enriched with 5 μg/mL GO used for the roughness indexes calculation

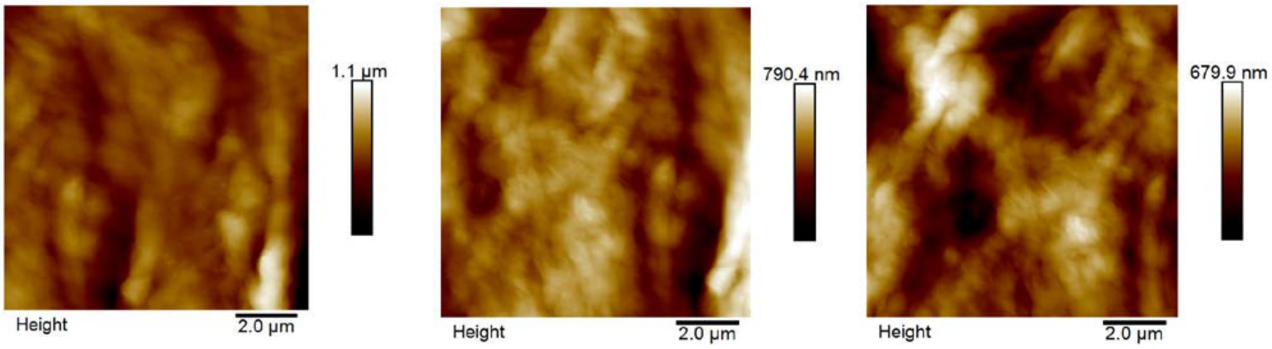

Figure S9. Height panels of bare Lamina enriched with 5 μg/mL GO sample used for the calculation of roughness indexes.

Table S2. Roughness indexes recovered from Panels of Figure S9 for Lamina enriched with 5 μg/mL GO sample.

Roughness recovered	Ra	Rq	Rmax	Sdq	Sdr
Panel 1	159 nm	200 nm	1239 nm	23.4 °	8.32 %
Panel 2	166 nm	208 nm	1257 nm	20.7 °	6.72 %
Panel 3	200 nm	240 nm	1414 nm	20.1 °	6.44 %

S2.6 Original AFM micrographs of Laminas enriched with 5 μg/mL GO used for the roughness indexes calculation

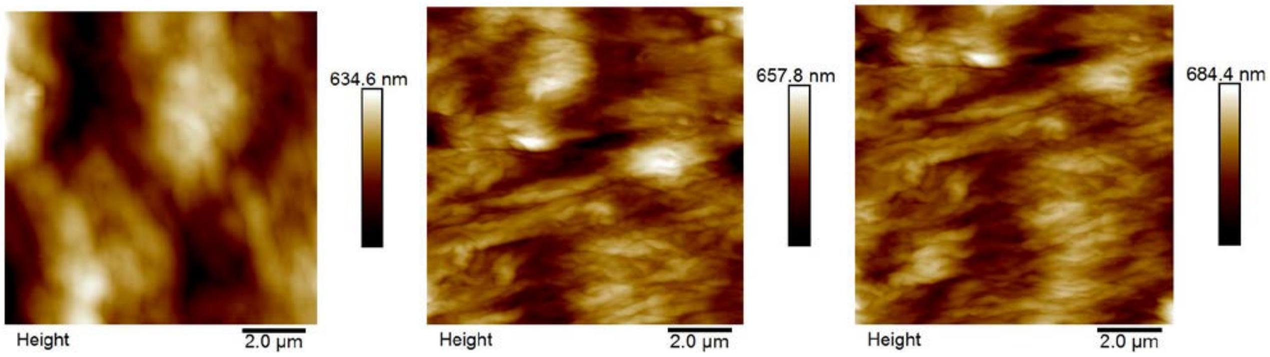

Figure S10. Height panels of bare Laminas enriched with 10 μg/mL GO sample used for the calculation of roughness indexes.

Table S3. Roughness indexes recovered from Panels of Figure S10 for Lamina enriched with 10 μg/mL GO sample.

Roughness recovered	Ra	Rq	Rmax	Sdq	Sdr
Panel 1	169 nm	206 nm	996 nm	13.3 °	2.72 %
Panel 2	258 nm	316 nm	1540 nm	32.3 °	16.1 %
Panel 3	190 nm	242 nm	1397 nm	32.1 °	15.8 %
